# Migratory Birds Facilitate the Spread of Multidrug‐Resistant Pathogenic *Escherichia coli* in Tanguar Haor of Bangladesh

**DOI:** 10.1111/1758-2229.70344

**Published:** 2026-04-12

**Authors:** Most Nahida Khatun, Sourav Chakraborty, Taslima Akter, Junaid Sarker Ifte, Jahan Ara Begum, Md Alimul Islam, Rokshana Parvin, Muhammad Tofazzal Hossain, Emdadul Haque Chowdhury

**Affiliations:** ^1^ Department of Pathology Faculty of Veterinary Science, Bangladesh Agricultural University Mymensingh Bangladesh; ^2^ Department of Microbiology and Hygiene Faculty of Veterinary Science, Bangladesh Agricultural University Mymensingh Bangladesh

**Keywords:** avian pathogenic *Escherichia coli*, dissemination of AMR, MDR, migratory bird, wetland ecosystem

## Abstract

This study investigated the occurrence of pathogenic and multidrug‐resistant (MDR) *Escherichia coli* in migratory birds inhabiting wetland habitats. A total of 167 freshly voided faecal samples were collected from migratory birds during the winters of 2023 and 2024. Isolation and identification of *E. coli* were performed using standard cultural and molecular techniques. Antimicrobial susceptibility testing was performed against 19 antibiotics, followed by the detection of resistance genes. Overall, the *E. coli* detection rate was 68.62% and 46.15% in 2023 and 2024, respectively. Multiple diarrheagenic *E. coli* pathotypes (ETEC, EAEC, EHEC, EPEC and EIEC) were identified, where the isolates from 2023 showed greater diversity. Most of the *E. coli* isolates were identified as MDR, with MDR patterns being more frequent in 2024. Resistance genes associated with tetracycline, β‐lactam and aminoglycoside antibiotics, where *tet*B, *tet*O, *tet*C, *bla*_CTX_, *bla*_SHV_ and *bla*_TEM_ were more prevalent than the rest of the genes. Our findings indicate that migratory birds are potential mobile reservoirs and disseminators of MDR *E. coli* within the wetland ecosystems, warranting coordinated and sustained One Health surveillance across environmental, wildlife and public health.

## Graphical abstract

Transmission dynamics of multidrug‐resistant pathogenic *Escherichia coli* in Tanguar Haor.

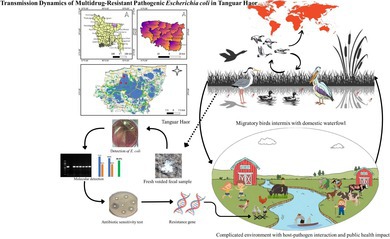

## 1 Introduction

Bangladesh attracts large numbers of migratory and wild birds during the winter months (November to February) to its wetland habitats. Billions of birds migrate annually, covering vast distances across continents (Guenther et al. 2012). It was reported that Bangladesh hosts approximately 712 species of birds, of which approximately 320 are migratory birds, as the country lies along two major migratory bird flyways in the East Asian–Australasian Flyway and the Central Asian Flyway (Parvin et al. 2014). Antimicrobial resistance (AMR) has emerged as a critical global issue within the One Health paradigm. Migratory birds, through their extensive movements and interactions with various habitats, may serve as carriers of AMR bacteria, facilitating their dissemination over long distances. During migration, these birds may intermingle with native birds, and environmental sources, such as contaminated feed and water, can facilitate the acquisition of resistant bacteria (Islam et al. 2021). Subsequently, the birds shed those bacteria through their faeces into aquatic systems, such as rivers, lakes, ponds and labour (Rashid et al. 2015; Islam et al. 2021). Previous studies have documented the role of migratory birds in transferring antibiotic‐resistant and/or multidrug‐resistant (MDR) bacteria (Atterby et al. 2016; Najdenski et al. 2018; Ramey et al. 2018; Lin et al. 2020) and facilitating their spread across ecosystems (Allen et al. 2010; Bonnedahl and Järhult 2014; Oteo et al. 2018; Zurfluh et al. 2019).

Among the diverse bacteria transmitted by migratory birds, *Escherichia coli* ( *E. coli* ) is of particular concern. Whilst many *E. coli* strains are commensal inhabitants in the guts of birds (Rahman et al. 2020). Certain strains possess virulent factors, enabling them to cause disease. The pathotypes include enterotoxigenic *E. coli* (ETEC), enterohemorrhagic *E. coli* (EHEC), enteroinvasive *E. coli* (EIEC), enteropathogenic *E. coli* (EPEC), enteroaggregative *E. coli* (EAEC), diffusely adherent *E. coli* (DAEC), and cell‐detaching *E. coli* (Smith et al. 2007). Pathogenic *E. coli* can cause diverse infections in both animals and humans, including diarrhoea, urinary tract infections (Foxman 2010), respiratory illnesses and bloodstream infections (Hopkins et al. 2005).

*E. coli* is one of the most widely used model bacteria for studying the evolution and spread of AMR (Stedt et al. 2014; Chung et al. 2018). The emergence of MDR *E. coli* has become a global public health threat, particularly for low‐ and middle‐income countries (LMICs) in Africa and Asia, including Bangladesh (Tawyabur et al. 2020). If not contained by 2050, AMR is estimated to cause hundreds of millions of human deaths, severe financial losses and a significant fall in livestock production (Orubu et al. 2020). Along with phenotypic confirmation of antibiotic resistance, molecular detection of AMR genes has become a vital approach to understanding antibiotic resistance mechanisms. Studies have confirmed that *E. coli* isolated from diverse environmental sources, including wild avian species, contain resistance genes to commonly used antibiotics such as tetracycline, β‐lactams and aminoglycosides (Mutuku et al. 2022; Islam et al. 2023; Ahmed and Gulhan 2024). Migratory birds move extensively among biomes and can acquire and disseminate these genes over large geographic areas. Hence, detecting antibiotic‐resistant genes in *E. coli* from migratory birds is crucial to reveal the environmental burden of AMR and its potential impact on public health.

Despite the growing literature, studies on the prevalence and pathotypes of MDR *E. coli* in migratory birds in Bangladesh remain inadequate. Particularly, Tanguar Haor, the Ramsar site in Bangladesh, has not been thoroughly investigated for MDR *E. coli* pathotypes. We hypothesise that migratory birds visiting this wetland harbour diverse MDR *E. coli* pathotypes, serving as reservoirs of AMR. Therefore, this study aimed to investigate the prevalence, diversity of pathotypes, AMR profiles and associated resistance genes of *E. coli* isolated from migratory birds inhabiting Tanguar Haor across two consecutive migratory seasons (2023 and 2024). The findings are intended to provide insight into the role of migratory birds in the environmental dissemination of antimicrobial‐resistant avian pathogenic *E. coli* pathotypes, highlighting their potential impact on ecosystem health and public health within a One Health framework.

## 2 Materials and Methods

### 2.1 Area of Study and Sample Collection

**FIGURE 1:**
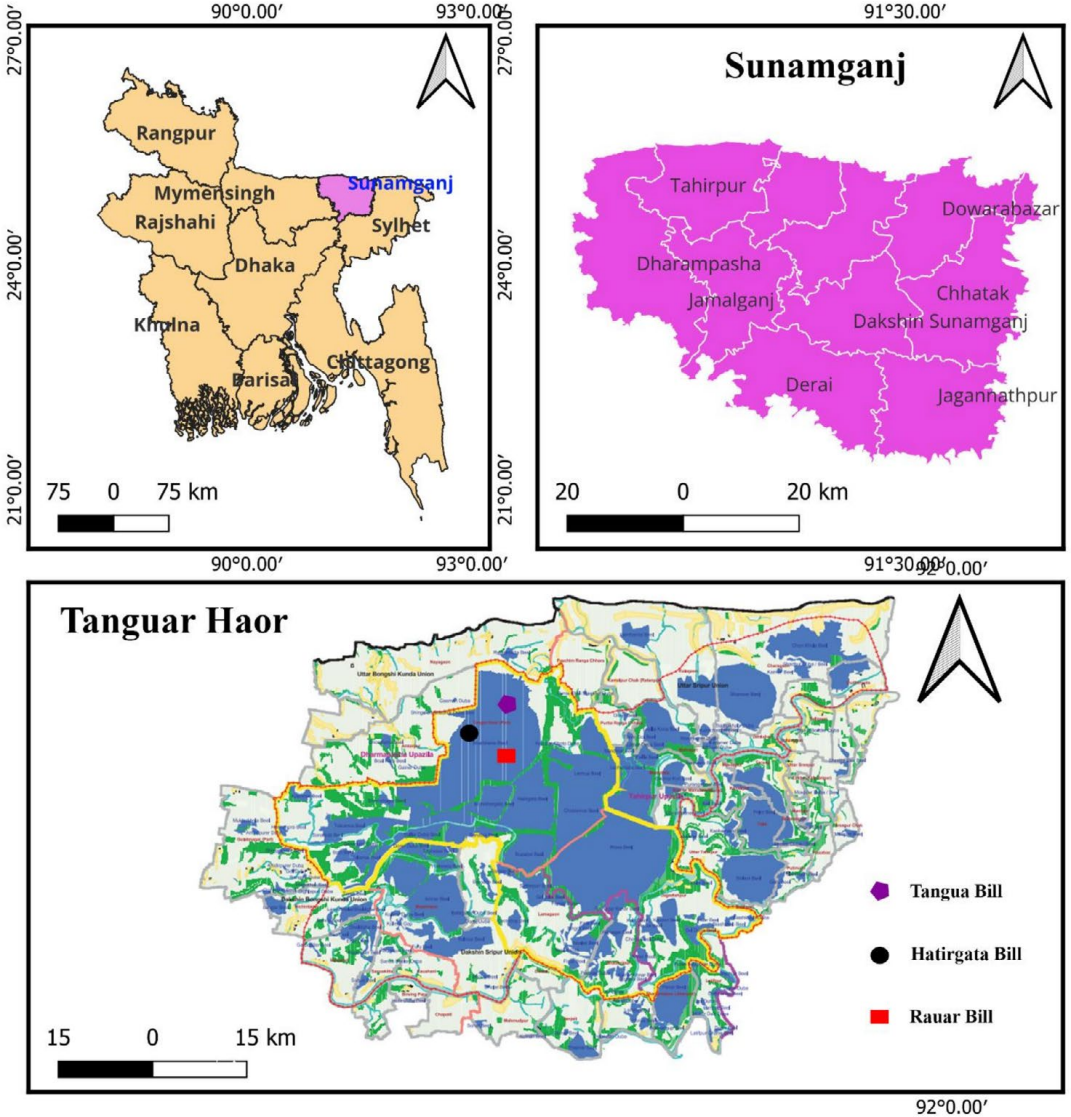
Demonstration of the study area (created with QGIS3.42.3). The purple colour denotes the sample collection area. The right panels, featuring a purple colour, display the upazila areas, while the lower panel, with a purple polygon, a black circle and a red rectangle, provides a closer view of the areas.

The study was conducted in Tanguar Haor, an ecologically important Ramsar wetland site situated in Sunamganj district, Bangladesh (Figure 1). This wetland is known for its high density of migratory birds during the winter season. In total, 167 freshly voided faecal samples were collected from migratory birds residing there between January 2023 and January 2024.

Fresh faecal droppings were collected early in the morning from grass blades and along the banks of lakes and rivers, ensuring minimal environmental exposure and maximum freshness (*Ethical approval: BAURES/ESRC/43/2024*). Approximately 1 g of faecal material was collected per sample using sterile disposable spoons and immediately transferred into Eppendorf tubes containing 1 mL of nutrient broth (HiMedia, India). The Eppendorf tubes were placed in sterile cooling boxes maintained at 4°C and then transported to the Department of Microbiology and Hygiene. Immediately upon arrival, all Eppendorf tubes containing faecal samples were incubated aerobically overnight at 37°C.

### 2.2 Isolation and Identification of *E. coli*

To isolate *E. coli* , enriched faecal samples were streaked onto Eosin Methylene Blue (EMB) agar plates using sterile inoculating loops and incubated overnight at 37°C. Colonies showing a characteristic green metallic sheen on EMB agar plates were presumptively identified as *E. coli* . These colonies were sub‐cultured to obtain pure colonies. For molecular confirmation of *E. coli* , DNA was extracted according to previously described methods (Sobur et al. 2019; Tawyabur et al. 2020). Briefly, 1 mL of pure culture was centrifuged at 5000 rpm for 5 min (ESCO, Indonesia). The pellet was then resuspended in 200 μL of phosphate‐buffered saline (PBS), boiled for 10 min, ice‐cooled for 7 min and then centrifuged again at 10,000 rpm for 10 min. The supernatant containing genomic DNA was collected and stored at −20°C for further analysis. Molecular confirmation of *E. coli* was conducted by amplifying the *malB* promoter region using a PCR thermal cycler (ThermoFisher Scientific, USA) (Wang et al. 1997). The 25 μL reaction mixture contained 12.5 μL of master mix (Promega, Madison, WI, USA), 1 μL each of forward and reverse primers, 7.5 μL of nuclease‐free water and 3 μL of template DNA. PCR products were resolved on a 1.5% agarose gel stained with 1% ethidium bromide and electrophoresed at 100 Volts for 30 min (Mini‐ES2, Allsheng, China). Bands were visualised using a UV transilluminator (BIO‐RAD, USA). A 100 bp DNA ladder (Promega, Madison, WI, USA) was used to confirm the amplicon's size.

### 2.3 Detection of *E. coli* Pathotypes

Five major pathotypes: *EPEC*, *EHEC*, *ETEC*, *EAEC* and *EIEC* were identified using multiplex PCR, following the protocol of Hegde et al. (2012). Before pathotype‐specific screening, the presence of the *chu*A virulence gene was detected by PCR using specific primers (Clermont et al. 2000). The *chu*A gene was initially screened as a marker to identify potentially pathogenic lineages before detailed pathotype‐specific gene analysis. Details of the primers and their corresponding amplicon sizes are provided in Table 1.

**TABLE 1:** Primers used in this study for the detection of *E. coli* and its pathotypes.

Organism	Target genes	Sequence	Size (bp)	AT (°C)	References
*E. coli*	*malB*	F: 5′‐GACCTCGGTTTAGTTCACAGA‐3′ R: 5′‐CACACGCTGACGCTGACCA‐3′	585	50°C	Wang et al. (1997)
PEC	*chu*A	F: 5′‐GACGAACCAACGGTCAGGAT‐3′ R: 5′‐TGCCGCCAGTACCAAAGACA‐3′	279	55°C	Clermont et al. (2000)
EPEC	*eaeA*	F: TGATAAGCTGC AGTCGAATCC R: CTGAACCAGATCGT AACGGC	229	57°C	Hegde et al. (2012)
	*bfpA*	F: CACCGTTACCGCAGGTGTGA R: GTTGCCGCTTCAGCAGGAGT	450		
EIEC	*ial*	F: CTGGTAGGTATGGTGAGG R: CCAGGCCAACAATTATTTCC	320		
ETEC	*elt*	F: CTCTATGTGCACACGGAGC R: CCATACTGATTGCCGCAAT	322	55°C	
	*Stla*	F: TCTTTCCCCTCTTTTAGTCAGTC R: CCGCACAGGCAGGATTAC	170		
EAEC	*CVD*432	F: CTGGCGAAAGACTGTATCAT R: CAATGTATAGAAATCCGCTGTT	630		
EHEC	*hlyA*	F: GCATCATCAAGCGTACGTTCC R: AATGAGCCAAGCTGGTTAAGCT	534		

Abbreviations: AT, annealing temperature; EAEC, enteroaggregative *E. coli* ; EHEC, enterohemorrhagic *E. coli* ; EIEC, enteroinvasive *E. coli* ; EPEC, enteropathogenic *E. coli* ; ETEC, enterotoxigenic *E. coli* ; PEC, pathogenic *E. coli* .

### 2.4 Antibiotic Susceptibility Testing (AST)

The antibiotic susceptibility of confirmed *E. coli* isolates was determined using the Kirby–Bauer disc diffusion method (Bauer et al. 1966) on Mueller–Hinton agar (MH agar; HiMedia, India), where *E. coli* ATCC 25922 was used as a quality control strain. Briefly, freshly cultured bacteria were suspended in phosphate‐buffered saline (PBS) and adjusted to a 0.5 McFarland standard (DEN‐1, Biosan, Latvia) and then uniformly spread out on MH agar plates. A total of 19 commonly used antibiotics of 8 classes were tested: aminoglycosides (amikacin‐30 μg, gentamicin‐10 μg, streptomycin‐10 μg), tetracyclines (oxytetracycline‐30 μg, doxycycline‐30 μg), β‐lactams (cephalosporins [cefixime‐5 μg, ceftriaxone‐30 μg, cefoxitin‐30 μg, cefalexin‐30 μg, cefuroxime‐30 μg], carbapenems [imipenem‐10 μg, meropenem‐10 μg], penicillins [amoxicillin‐10 μg, amoxicillin‐clavulanic acid‐30 μg]), macrolides (azithromycin‐15 μg), fluoroquinolones (nalidixic acid‐30 μg, ciprofloxacin‐5 μg, levofloxacin‐5 μg), phenicol (chloramphenicol‐30 μg) and phosphonic (fosphomycin‐50 μg) (Oxoid, UK). Zones of inhibition were measured in millimetres and interpreted as resistant, intermediate, or sensitive according to the Clinical and Laboratory Standards Institute (CLSI) guidelines (CLSI 2023). Isolates resistant to ≥ 3 antibiotic classes were defined as MDR, as defined by (Magiorakos et al. 2012).

### 2.5 Detection of Antibiotic‐Resistant Genes by PCR

Tetracycline‐resistant genes *tet*A, *tet*B, *tet*C, *tet*D, *tet*E, *tet*K, *tet*M and *tet*O; β‐lactam‐resistant genes, including *bla*_CTX‐M3_, *bla*_TEM‐1_ and *bla*_SHV_; aminoglycoside‐resistant gene *aacC2* were identified using standard PCR protocols (Ng et al. 2001) (Table 2).

**TABLE 2:** List of the primers used for the detection of tetracycline, β‐lactam and aminoglycoside‐resistant genes in *E. coli* isolates.

Group of antibiotics	Target gene	Primer sequences (5′‐3′)	Amplicon size (bp)	References
Tetracycline	*tet*A	F: GGTTCACTCGAACGACGTCA	210	Ng et al. (2001)
		R: CTGTCCGACAAGTTGCATGA		
	*tet*B	F: TTG GTT AGG GGC AAG TTT TG	659	
		R: GTA ATG GGC CAA TAA CAC CG		
	*tet*C	F: CTT GAG AGC CTT CAA CCC AG	418	
		R: ATG GTC GTC ATC TAC CTG CC		
	*tet*D	F: AAA CCA TTA CGG CAT TCT GC	787	
		R: GAC CGG ATA CAC CAT CCA TC		
	*tet*E	F: AAA CCA CAT CCT CCA TAC GC	287	
		R: AAA TAG GCC ACA ACC GTC AG		
	*tet*K	F: TTA GGT GAA GGG TTA GGT CC	169	
		R: GCA AAC TCA TTC CAG AAG CA		
	*tet*M	F: GTG GAC AAA GGT ACA ACG AG	406	
		R: CGG TAA AGT TCG TCA CAC AC		
	*tet*O	F: ACG GAR AGT TTA TTG TAT ACC	515	
		R: TGG CGT ATC TAT AAT GTT GAC		
β‐lactam	*bla*_CTX‐M3_	F: GAAGGTCATCAAGAAGGTGCG	560	Pandit et al. (2020)
		R: GCATTGCCACGCTTTTCATAG		
	*bla*_TEM‐1_	F: ATAAAATTCTTGAAGACGAAA	1080	Yao et al. (2007)
		R: GACAGTTACCAATGCTTAATC		
	*bla*_SHV_	F: GGGTTATTCTTATTTGTCGC	930	Dutta et al. (2013)
		R: TTAGCGTTGCCAGTGCTC		
Aminoglycoside	*aacC2*	F: GGCAATAACGGAGGCAATTCGA	450	Chen et al. (2004)
		R: CTCGATGGCGACCGAGCTTCA		

### 2.6 Statistical Analysis

The results were organised using Microsoft Excel 2019. Spearman's rank correlation coefficient was calculated using the Analysis ToolPak add‐in in Excel to assess the relationships between AMR patterns of *E. coli* pathotypes. The Chi‐square test of independence was performed using IBM SPSS Statistics for Windows, version 29.0 (IBM Corp., Armonk, NY, USA) to determine significant associations of *E. coli* isolation in two migratory seasons. A *p* < 0.05 was considered statistically significant.

## 3 Results

### 3.1 Occurrence of *E. coli*

A total of 167 faecal samples were collected from Tanguar Haor across two consecutive years: January 2023 (*n* = 102) and January 2024 (*n* = 65). In 2023, 70 (68.62%) isolates were confirmed as *E. coli* through PCR among the 76 culture‐positive isolates. On the other hand, in 2024, only 30 out of 65 samples (46.15%) tested positive by culture and PCR. Overall, 100 (59.88%) of the 167 samples were confirmed as *E. coli* . The *p*‐value for the differences in the occurrence of *E. coli* isolates between January 2023 and January 2024 was statistically significant (*p* = 0.0064; *p* < 0.05), indicating that the proportion of *E. coli* positive isolates in January 2023 (68.62%) is significantly higher than in January 2024 (46.15%) (Table 3).

**TABLE 3:** Comprehensive sampling and detection summary of *E. coli* isolates obtained from the Tanguar Haor in Bangladesh.

Sampling area	Year	Samples (*n*)	Culture positive (%)	PCR positive (%)	Overall isolation (%)	*p*
Tanguar Haor	Jan‐23	102	76 (74.50)	70 (68.62)	100 (59.88%)	0.0064^a^
	Jan‐24	65	30 (46.15)	30 (46.15)		

Abbreviation: Jan, January.

^a^ A *p*‐value less than 0.05 (*p* < 0.05) was considered significant.

### 3.2 Presence of *E. coli* Pathotypes

The presence of pathogenic *E. coli* was initially detected by amplifying the *chu*A gene. It was revealed that 45 isolates (44.11%) and 8 isolates (12.30%) tested positive for *chu*A in 2023 and 2024, respectively. After that, pathotype‐specific PCR analysis identified the ETEC in 36 isolates (35.29%), EAEC in 15 (14.70%), EHEC in 9 (8.82%), EPEC in 39 (38.23%) and EIEC in 31 isolates (30.39%) in the samples of 2023. In contrast, among the samples collected in 2024, ETEC and EPEC were detected in only five isolates (7.69%), while EHEC was found in just one isolate (1.53%). No isolates tested positive for EAEC or EIEC in the samples. The statistical evaluation indicates significant differences in the prevalence of *chu*A (*p* < 0.0001), ETEC (*p* = 0.0001), EAEC (*p* = 0.0046), EPEC (*p* < 0.0001) and EIEC (*p* < 0.0001) between the 2 years. Nevertheless, the difference in EHEC prevalence was not statistically significant (*p* = 0.111). Table 4 summarises the overview of the pathotypes along with their *p*‐value and significance.

**TABLE 4:** Summary of *E. coli* pathotypes isolated from the Tanguar Haor in Bangladesh.

Parameters	Tanguar Haor‐‘23 (*n* = 102)	Tanguar Haor‐ ‘24 (*n* = 65)	*χ* ^2^ value	*p*	Significance
*chu*A	45 (44.11%)	8 (12.30%)	20.901	< 0.0001^a^	Yes
ETEC	36 (35.29%)	5 (7.69%)	15.228	0.0001^a^	Yes
EAEC	15 (14.70%)	0 (0%)	8.042	0.0046^a^	Yes
EHEC	9 (8.82%)	1 (1.53%)	2.544	0.111	No
EPEC	39 (38.23%)	5 (7.69%)	23.28	< 0.0001^a^	Yes
EIEC	31 (30.39%)	0 (0%)	22.10	< 0.0001^a^	Yes

Abbreviations: EAEC, enteroaggregative *E. coli* ; EHEC, enterohemorrhagic *E. coli* ; EIEC, enteroinvasive *E. coli* ; EPEC, enteropathogenic *E. coli* ; ETEC, enterotoxigenic *E. coli* .

^a^ A *p*‐value of less than 0.05 (*p* < 0.05) was considered significant.

### 3.3 Comparison of Antibiogram Profiles Between Two Migration Seasons

Notable differences were observed in the antibiotic resistance patterns of *E. coli* isolates from Tanguar Haor across two consecutive winter seasons. All isolates (100%) exhibited resistance to amoxicillin (AML) in both study years. The resistance rate for amoxicillin‐clavulanic acid (AMC) rose by more than 30% (from 54.28% to 86.66%). The resistance rate against nalidixic acid (NA) surged significantly from 18.57% to 70%.

**FIGURE 2:**
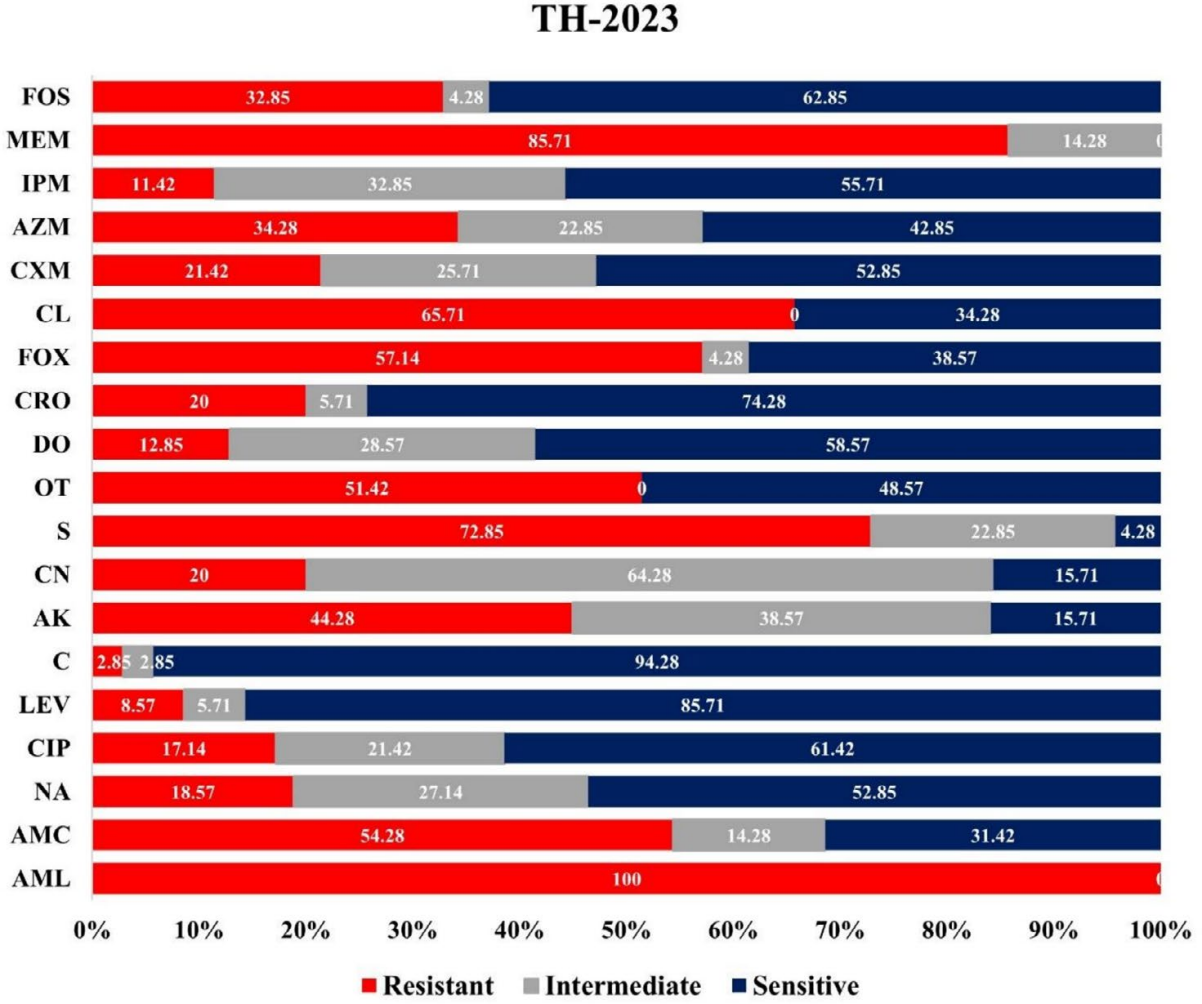
Antibiogram profiles of *E. coli* isolated from migratory birds of Tanguar Haor 2023. AK, amikacin; AMC, amoxicillin + clavulanic acid; AML, amoxicillin; AZM, azithromycin; C, chloramphenicol; CIP, ciprofloxacin; CL, cephalexin; CN, Gentamicin; CRO, ceftriaxone; CXM, cefuroxime; DO, doxycycline; FOS, fosfomycin; FOX, cefoxitin; IPM, imipenem; LEV, levofloxacin; MEM, meropenem; NA, nalidixic acid; OT, oxytetracycline; S, streptomyci.

**FIGURE 3:**
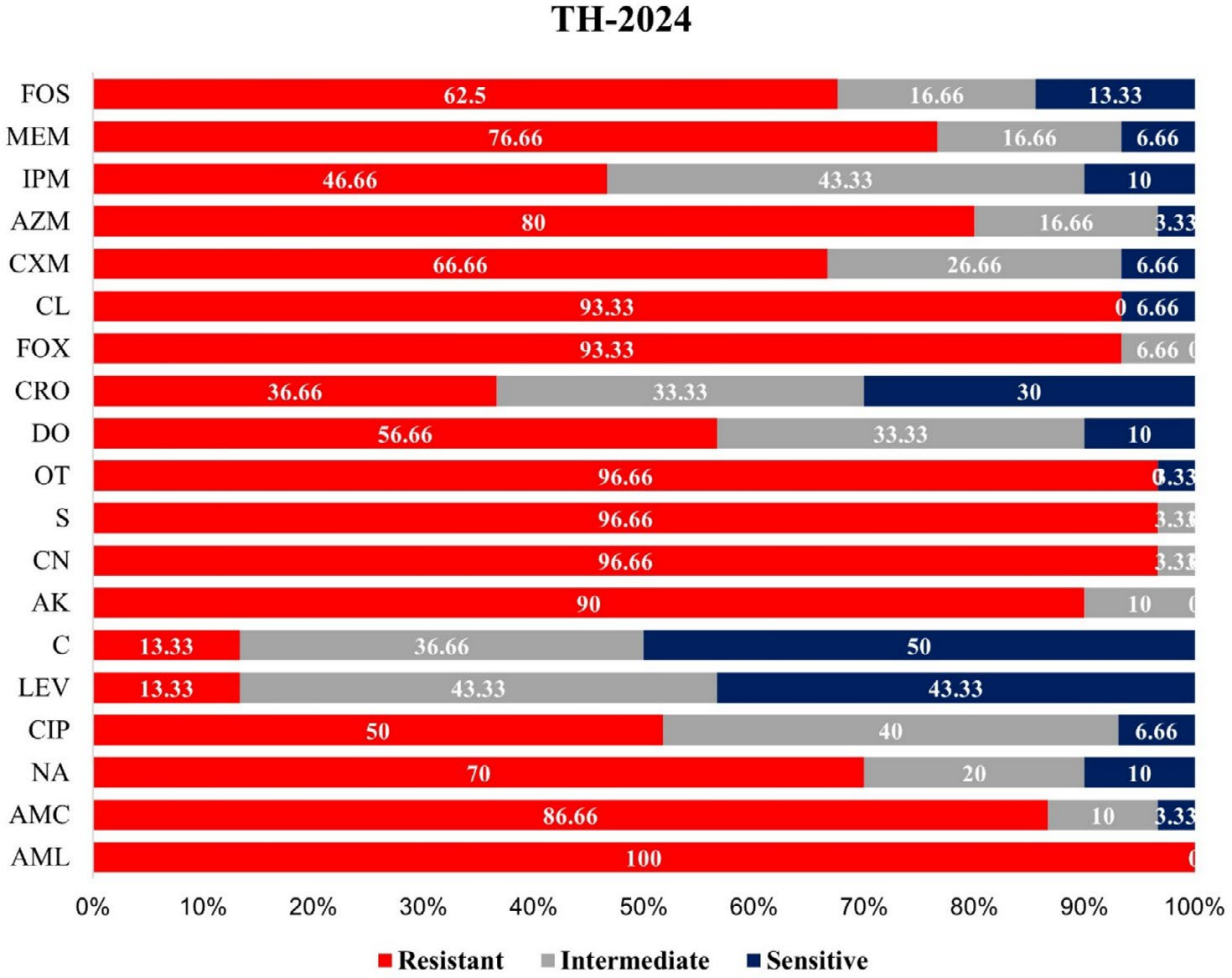
Antibiogram profiles of *E. coli* isolated from migratory birds of Tanguar Haor 2024. AK, amikacin; AMC, amoxicillin + clavulanic acid; AML, amoxicillin; AZM, azithromycin; C, chloramphenicol; CIP, ciprofloxacin; CL, cephalexin; CN, gentamicin; CRO, ceftriaxone; CXM, cefuroxime; DO, doxycycline; FOS, fosfomycin; FOX, cefoxitin; IPM, imipenem; LEV, levofloxacin; MEM, meropenem; NA, nalidixic acid; OT, oxytetracycline; S, streptomycin.

Resistance to ciprofloxacin (CIP) nearly tripled, from 17.14% to 50%. A modest increase in the resistance to levofloxacin (LEV) was observed, rising from 8.57% to 13.33%. Though isolates showed slight resistance to chloramphenicol (C), it increased from 2.85% to 13.33% in the year 2024. The resistance rate of amikacin (AK) doubled, from 44.28% to 90%. A dramatic rise in the resistance to gentamicin (CN) was observed, increasing from 20% to 96.66%. Resistance to streptomycin (S) increased from 72.85% to 96.66%, whereas resistance to oxytetracycline (OT) increased from 51.42% to 96.66%. A notable climb in the resistance was observed in the case of doxycycline (DO), rising from 12.85% to 56.66%. Resistance to ceftriaxone (CRO) increased from 12.85% to 56.66% (Figures 2 and 3).

The resistance rate of cefoxitin (FOX) increased from 57.14% to 93.33%. In the case of cloxacillin (CL), the resistance rate jumped from 65.71% to 93.33%. Resistance to cefuroxime (CXM) tripled, rising from 21.42% to 66.66%. For azithromycin (AZM), the resistance increased from 34.28% to 80%. A fourfold increase in resistance was observed for imipenem (IPM), increasing from 11.42% to 46.66%. Notably, among all the increments in the resistance for the rest of the antibiotics, the resistance rate to meropenem (MEM) decreased slightly from 85.71% to 76.66%. Resistance to fosfomycin (FOS) increased from 32.85% to 62.5% (Figures 2 and 3).

### 3.4 Status of the MAR (Multiple Antibiotic Resistance) Index

In 2023, 49 out of 70 (70%) *E. coli* isolates were identified as MDR based on a MAR index of ≥ 0.3. In 2024, all 30 *E. coli* isolates (100%) were MDR. The MAR index among 2023 isolates ranged from 0.105 to 0.78, whereas the range increased from 0.47 to 0.89 for the 2024 isolates. In 2023, the highest MAR index value recorded was 0.78, corresponding to an *E. coli* isolate that was resistant to 15 antibiotics across 8 different classes.

This isolation was confirmed to harbour enterotoxigenic and enteropathogenic strains. Additionally, one isolate showed a MAR index of 0.73, with resistance to 14 antibiotics across 6 antibiotic classes; this isolate carried all five *E. coli* pathotypes. The remaining MDR isolates of 2023 also contained various combinations of pathotypes, as detailed in Table S1. In 2024, the highest observed MAR value was 0.89, 0.89, corresponding to an *E. coli* isolate that was resistant to 17 antibiotics across 7 different classes. The MAR value was observed to be 0.84 in three isolates, which were resistant to 16 antibiotics across 7 classes. However, an enteropathogenic strain was detected in one isolate, and no pathotypes were detected in the other two isolates. Nevertheless, several other MDR isolates from 2024 did confirm the presence of different pathotypes (Table S2).

### 3.5 Pearson Correlation Coefficients for Pairs of Antibiotics

**FIGURE 4:**
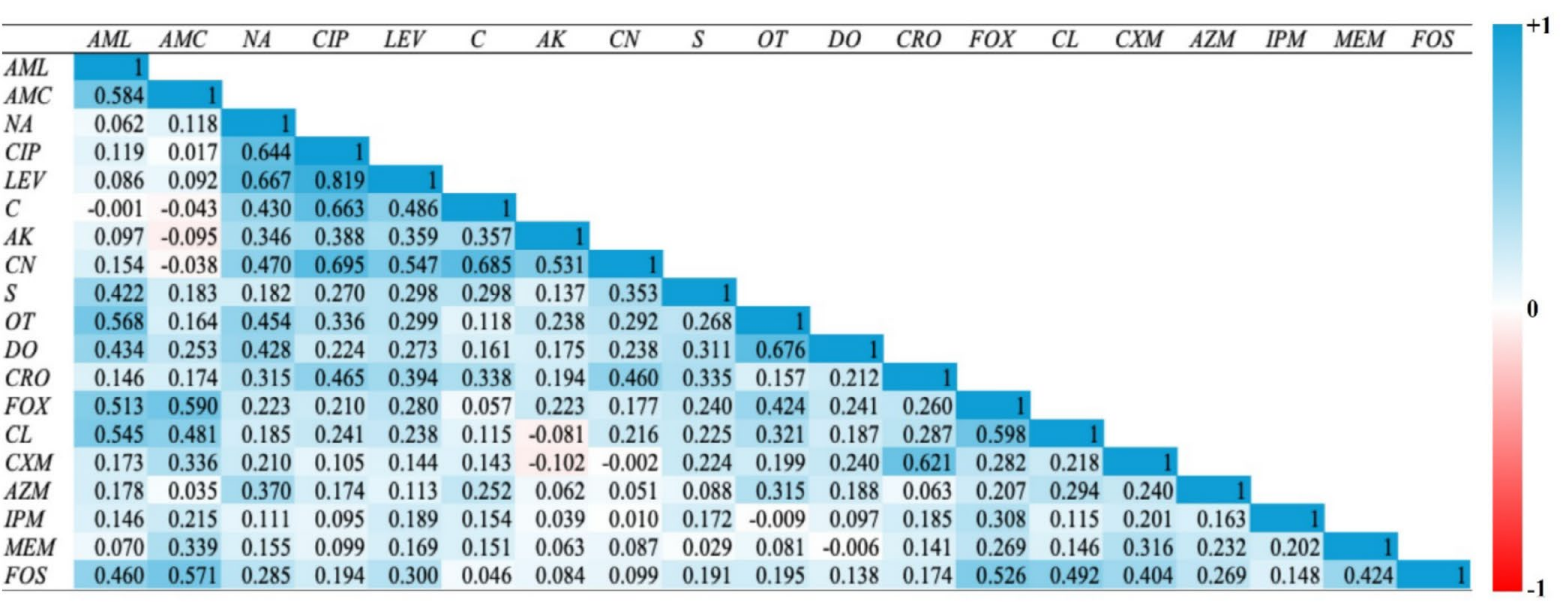
The Spearman coefficient matrix shows pairwise relationships among antibiotic resistance profiles of *E. coli* isolated from Tanguar Haor in 2023. Colour intensity represents the strength and direction of the correlation (*ρ*). Blue cells and values closer to +1 mean two antibiotics behave very similarly (bacteria sensitive to one are usually sensitive to the other). Red cells and values closer to −1 mean the antibiotics tend to work in opposite ways (if bacteria are sensitive to one, they're often resistant to the other). White or pale cells and values near 0 mean there is little or no relationship between the two antibiotics.

**FIGURE 5:**
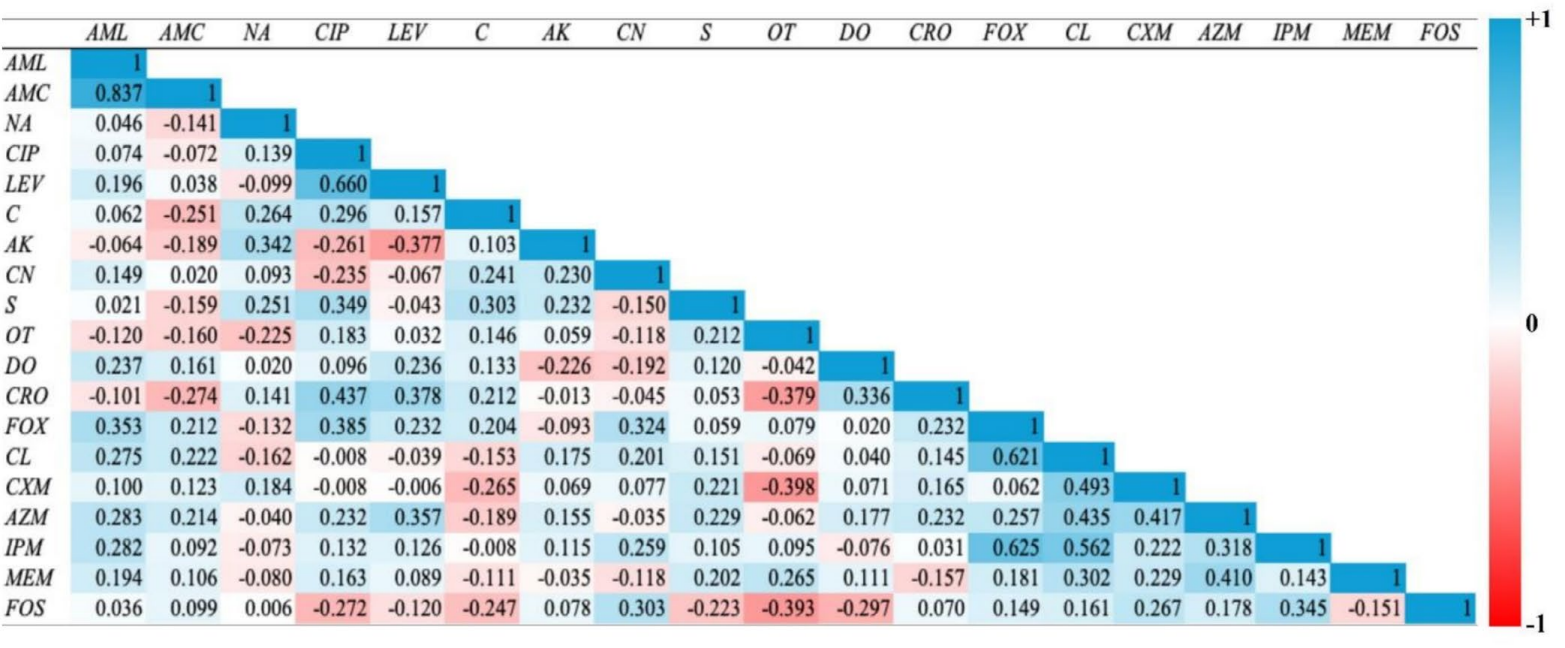
The Spearman coefficient matrix shows pairwise relationships among antibiotic resistance profiles of *E. coli* isolated from Tanguar Haor in 2024. Colour intensity represents the strength and direction of the correlation (*ρ*). Blue cells and values closer to +1 mean two antibiotics behave very similarly (bacteria sensitive to one are usually sensitive to the other). Red cells and values closer to −1 mean the antibiotics tend to work in opposite ways (if bacteria are sensitive to one, they're often resistant to the other). White or pale cells and values near 0 mean there is little or no relationship between the two antibiotics.

The Pearson rank correlation analysis was performed to assess AMR using the inhibition zone diameter for each antibiotic to identify co‐resistance patterns. *E. coli* isolates of 2023 showed a significant positive correlation between several antibiotic pairs. Notably, LEV and CIP (*ρ* = 0.819), CN and CIP (*ρ* = 0.695), suggested the possibility of cross‐resistance and shared resistance mechanisms. Moreover, strong correlations were observed for FOX and CL (*ρ* = 0.598) and FOX and CRO (*ρ* = 0.260) (Figure 4 and Table S3). On the other hand, *E. coli* isolates of 2024 produced variable correlation patterns. Compared to the previous year, during 2024, some antibiotic pairs retained a strong correlation (CIP and LEV, *ρ* = 0.660). In the meantime, several other antibiotics displayed negative or weak correlations (Figure 5 and Table S4).

### 3.6 Detection of Antibiotic‐Resistant Genes (ARGs) From the *E. coli* Isolates

Of the tetracycline‐resistant genes, *tet*B, *tet*O and *tet*C showed significant differences between the 2 years (2023 and 2024). Among them, the detection rate of *tet*B was significantly higher in 2024 (43.3%) compared to 2023 (11.4%) (*χ* ^2^ = 11.034, *p* = 0.0009). On the other hand, *tetO* showed a significantly higher detection in 2023 (57.1%) than in 2024 (23.3%) (*χ* ^2^ = 8.327, *p* = 0.0039). This study found that *tet*C was more prevalent in 2023 (68.6%) than in 2024 (46.7%), but the difference was not statistically significant (*χ* ^2^ = 3.398, *p* = 0.0653). No statistically significant differences were observed in the detection rates of *tet*A, *tet*D, *tet*E, *tet*K and *tet*M between the 2 years. The difference in *bla*_TEM_ prevalence (68.6% in 2023 and 46.7% in 2024) was marginally non‐significant (*χ* ^2^ = 3.398, *p* = 0.0653). The *bla*_CTX_ gene was detected in 34.3% of isolates in 2023, compared to only 10.0% in 2024 (*χ* ^2^ = 5.112, *p* = 0.0238). Additionally, the detection of *bla*_SHV_ dropped from 57.1% in 2023 to 26.7% in 2024 (*χ* ^2^ = 6.641, *p* = 0.0100). No statistically significant difference was observed in the detection rate of *aacC*2 (aminoglycoside resistance gene) between the two seasons (Table 5).

**TABLE 5:** Isolation rates of tetracycline, β‐lactam and aminoglycoside‐resistant genes (ARGs) from the *E. coli* isolates.

Group of antibiotics	Name the antibiotic resistance genes	Isolation rate in 2023	95% Confidence interval	Isolation rate in 2024	95% Confidence interval	Chi‐square	*p*
Tetracycline	*tet*A	57.1% (40/70)	45.5%–68.1%	43.3% (13/30)	27.4%–60.8%	1.101	0.2940
	*tet*B	11.4% (8/70)	5.9%–21.0%	43.3% (13/30)	27.4%–60.8%	11.034	0.0009^a^
	*tet*C	68.6% (48/70)	57.0%–78.2%	46.7% (14/30)	30.2%–63.9%	3.398	0.0653
	*tet*D	34.3% (24/70)	24.2%–46.0%	20.0% (6/30)	9.5%–37.3%	1.417	0.2339
	*tet*E	0.0% (0/70)	0.0%–5.2%	3.3% (1/30)	0.6%–16.7%	0.192	0.6609
	*tet*K	0.0% (0/70)	0.0%–5.2%	3.3% (1/30)	0.6%–16.7%	0.192	0.6609
	*tet*M	34.3% (24/70)	24.2%–46.0%	16.7% (5/30)	7.3%–33.6%	2.368	0.1238
	*tet*O	57.1% (40/70)	45.5%–68.1%	23.3% (7/30)	11.8%–40.9%	8.327	0.0039^a^
β‐lactam	*bla*_TEM_	68.6% (48/70)	57.0%–78.2%	46.7% (14/30)	30.2%–63.9%	3.398	0.0653
	*bla*_CTX_	34.3% (24/70)	24.2%–46.0%	10.0% (3/30)	3.5%–25.6%	5.112	0.0238^a^
	*bla*_SHV_	57.1% (40/70)	45.5%–68.1%	26.7% (8/30)	14.2%–44.4%	6.641	0.0100^a^
Aminoglycoside	*aacC*2	34.3% (24/70)	24.2%–46.0%	26.7% (8/30)	14.2%–44.4%	0.265	0.6068

^a^ A *p*‐value less than 0.05 (*p* < 0.05) was considered significant.

## 4 Discussion

*E. coli* has been well‐documented to have the capacity to develop and spread AMR (Ibrahim et al. 2016; Islam et al. 2021). This study aimed to examine the presence of pathogenic, MDR *E. coli* in the faecal samples of migratory birds in Tanguar Haor, Bangladesh, an internationally recognised Ramsar site. Our findings indicate that wetlands function as hubs, with migratory birds serving as mobile vectors of MDR *Escherichia coli* , thereby contributing to the global dissemination of AMR.

A notable difference was observed in the isolation rates of *E. coli* , where the occurrence reached 68.62% in 2023, but by 2024, it had dropped to 46.15%. The variation between years may reflect differences in environmental parameters (water quality, temperature and level of faecal contamination in the wetland ecosystem). These parameters can influence the persistence, proliferation and transmission of enteric bacteria in aquatic environments (Sidhu and Toze 2009; Jang et al. 2017). It is plausible that elevated human and livestock activity in and around the wetland can contribute to a higher bacterial load, as discussed in earlier studies (Díaz et al. 2010), which may have happened during 2023. Furthermore, annual fluctuations in the migratory bird population, species diversity and stopover durations in the staging areas may have influenced the pathogen transmission dynamics (Waldenström et al. 2002; Hubalek 2004).

The overall occurrence of *E. coli* across two consecutive winters at Tanguar Haor was 59.88%, lower than the occurrence reported in a previous study conducted in the same area (Rahman et al. 2024) and below the occurrence rates reported in other parts of Bangladesh, where values as high as 83% have been documented (Islam et al. 2021; Neloy et al. 2024). In contrast, the isolation rate found in this study is substantially higher than that reported from other countries, such as 33.3% in Egypt (Fahim et al. 2019) and 33.9% in Italy (Dotto et al. 2016). Considering that *E. coli* is a common intestinal commensal in birds, its frequent detection in migratory bird faeces is expected. Nevertheless, the long‐distance migrations of these birds expose them to diverse ecological niches and feeding habitats, which can affect the acquisition, persistence and dissemination of various *E. coli* strains, including those with pathogenic or antimicrobial‐resistant characteristics.

This study provides the first evidence of avian‐associated pathogenic *E. coli* pathotypes (APEC) in migratory birds in Bangladesh. The presence of these pathotypes varied across two consecutive years, as discussed earlier, likely reflecting the influence of environmental conditions and anthropogenic activities in the study areas. Importantly, APEC is not only linked to a wide spectrum of diseases in birds but also carries significant zoonotic potential with transmission routes extending to humans, livestock and other avian species (Camarda et al. 2007), being implicated in gastroenteritis, urinary tract infections, neonatal meningitis and, in severe cases, haemorrhagic diarrhoea and haemolytic uremic syndrome (Liu 2015). Furthermore, it should be noted that even non‐pathogenic *E. coli* isolates may acquire virulence through continuous passage and genetic adaptation under selective pressures, thereby evolving into pathogenic forms (Ochman et al. 2000; Wassenaar and Gaastra 2001). These findings underscore the importance of migratory birds as reservoirs and potential disseminators of pathogenic *E. coli* , with implications for both avian and public health.

Migratory birds serve not only as biological carriers but also as amplifying hosts for MDR (Allen et al. 2010; Mellata 2013; Najdenski et al. 2018). This study confirmed the presence of MDR *E. coli* strains among avian isolates, consistent with earlier studies (Blanco et al. 2007; Najdenski et al. 2018). We tested 19 antibiotics of nine different classes, and it was observed that isolates showed a higher level of resistance in both years. Even the MAR index increased in 2024 compared to 2023, where 70% of the isolates showed a MAR index of 0.3 in 2023, and 100% of the isolates showed a MAR index > 0.3. MAR index > 0.3 is considered a MDR pathogen. Earlier studies also reported resistant *E. coli* in migratory birds, resistance against antibiotics such as ampicillin, ciprofloxacin, chloramphenicol, tetracycline, streptomycin and gentamicin (Literák et al. 2007; Foti et al. 2011; Shobrak and Abo‐Amer 2014; Ruzauskas and Vaskeviciute 2016; Ramey et al. 2018). Remarkably, resistance to Fosfomycin, a last‐resort antibiotic, was detected in > 85% of 2024 isolates, while over 60% of 2023 isolates showed sensitivity to Fosfomycin. The emergence of resistance to Fosfomycin is particularly alarming due to its critical importance in human medicine. Elevated MAR indices in *E. coli* isolated from migratory birds reflect a high risk to the local biodiversity and ecosystem health.

There were significant differences in the detection of *tet*B, *tet*O, *bla*_CTX_ and *bla*_SHV_, indicating substantial shifts in the transmission of resistant genes between 2023 and 2024. Other ARGs, including *tet*A, *tet*D, *tet*E, *tet*K, *tet*M, *bla*_TEM_ and *aacC*2, were also present in both migratory seasons. These findings align with previous studies that reported the presence of similar resistance genes in *E. coli* from poultry (Islam et al. 2023), wild birds (Nowaczek et al. 2021; Ahmed and Gulhan 2024) and migratory birds (Mohsin et al. 2017; Ramey et al. 2018; Islam et al. 2021; Yuan et al. 2021; Rahman et al. 2024). Numerous studies have reported MDR and extended‐spectrum β‐lactamase (ESBL)‐producing *E. coli* in wild birds (Athanasakopoulou et al. 2022), with varying resistance rates globally: 50.9% in Japan (Fukuda et al. 2021), 25% in Egypt (Tawakol and Younes 2019) and 62.19% in Turkey (Şahan Yapicier et al. 2022). Genes such as *aadA1*, *strA*, *strB* and *aac(3)‐II/IV* contribute to aminoglycoside resistance (Radhouani et al. 2009; Garneau‐Tsodikova and Labby 2016; Islam et al. 2022), while *tet* genes and efflux mechanisms mediate tetracycline resistance (Grossman 2016), and β‐lactamases drive β‐lactam resistance (Shaikh et al. 2015). Prevalence of tetracycline and ampicillin resistance was shown to be highly prevalent in avian *E. coli* (Elsohaby et al. 2021; Di Francesco et al. 2022).

The detection of MDR *E. coli* in migratory birds is consistent with previous reports from Bangladesh and other global regions (Hasan et al. 2014; Shobrak and Abo‐Amer 2014; Ramey et al. 2018), raising concerns regarding the long‐distance dissemination of AMR through avian migration (Fu et al. 2019). Cross‐species transmission of resistant bacteria from livestock to wild birds has also been documented (Kozak et al. 2009). In Bangladesh, domestic ducks commonly share water bodies with migratory birds during the day, and in the evening, these ducks intermingle with domestic poultry and livestock. Such interactions create ample opportunities for multidirectional exchange of MDR *E. coli* pathotypes, including possible human spillover, thereby elevating risks to public health and food safety. The main limitation of this study is that the faecal samples were not collected directly from birds due to their protected status. Although our approach has the possibility of introducing some environmental contamination, it allowed for ethical sampling. Additionally, while Tanguar Haor was accounted in our study for its high biodiversity, including other haor regions, which would provide more comprehensive data of MDR *E. coli* distribution among the migratory bird populations.

Addressing this complex challenge requires a coordinated, multi‐sectoral response that integrates awareness‐building, continuous surveillance, targeted research and the implementation of biosecurity measures around wetlands where migratory birds congregate in winter. As the issue spans human, animal and environmental health, a One Health‐oriented approach is essential.

## 5 Conclusions

This study provides the first comprehensive evidence of MDR pathogenic *Escherichia coli* in migratory birds from major wetland ecosystems of Bangladesh. Our findings highlight the role of migratory birds in disseminating pathogenic and non‐pathogenic MDR *E. coli* in the local environment, along with virulent and resistant genes during migration, raising significant concerns for human and animal health as well as food safety. The emergence of Fosfomycin resistance is particularly alarming. Future studies should expand surveillance to other wetlands in and outside of Bangladesh to assess the geographic and seasonal distribution of MDR *E. coli* and other potential zoonotic pathogens. Integrating epidemiological, metagenomic and ecological parameters within a One Health framework will support the development of effective strategies to fight against AMR.

## Author Contributions

**Most Nahida Khatun:** formal analysis, methodology, data curation, visualisation, writing‐original draft, writing‐review and editing. **Sourav Chakraborty:** formal analysis, methodology, data curation, visualisation, writing‐original draft, writing‐review and editing. **Taslima Akter:** formal analysis, methodology. **Junaid Sarker Ifte:** methodology. **Jahan Ara Begum:** writing‐review and editing. **Md Alimul Islam:** writing‐review and editing. **Rokshana Parvin:** formal analysis, data curation, supervision, writing‐review and editing. **Muhammad Tofazzal Hossain:** conceptualisation, investigation, methodology, data curation, funding acquisition, supervision, writing‐original draft, writing‐review and editing. **Emdadul Haque Chowdhury:** conceptualisation, investigation, data curation, formal analysis, supervision, writing‐review and editing.

## Funding

This work was supported by Bangladesh Agricultural University Research System (2024/161/BAU).

## Ethics Statement

The Bangladesh Agricultural University Research System's ethical committee has authorised the current study, which has permission number BAURES/ESRC/43/2024. The studies were carried out in compliance with institutional norms and local laws. The owners gave their written informed agreement for their animals to take part in this study.

## Conflicts of Interest

The authors declare no conflicts of interest.

## Supporting information


**Table S1:** Multidrug‐resistant patterns of the *E. coli* isolated from the Tanguar Haor in 2023.

**Table S2:** Multidrug‐resistant patterns of the *E. coli* isolated from the Tanguar Haor in 2024.

**Table S3:** Pearson correlation coefficient of antibiotics and antibiotics correlation with 95% significance in *E. coli* isolates from Tanguar Haor‐2023.

**Table S4:** Pearson correlation coefficient of antibiotics and antibiotics correlation with 95% significance in *E. coli* isolates from Tanguar Haor‐2024.

## Data Availability

The data that supports the findings of this study are available in the Supporting Information of this article.
