# Supplementary material for: Migratory Birds Facilitate the Spread of Multidrug‐Resistant Pathogenic Escherichia coli in Tanguar Haor of Bangladesh
Source: Environ Microbiol Rep. 2026 Apr 12;18(2):e70344. doi: 10.1111/1758-2229.70344 (PMC13070580; doi:10.1111/1758-2229.70344)
Supplement: Supplementary file 1 — Table S1: Multidrug‐resistant patterns of the E. coli isolated from the Tanguar Haor in 2023. [file EMI4-18-e70344-s001.docx]

**Supplementary table 1:** Multidrug-resistant patterns of the *E. coli* isolated from the Tanguar haor in 2023

| **Sample ID** | **Presence of Pathotypes** | **Multidrug-Resistance Patterns** | **No. of Resistant Antibiotics (Classes)** | **MAR (a/b)** | **Overall No. of MDR isolates (%)** |
| --- | --- | --- | --- | --- | --- |
| 62E | ET, EP | AMC, AML, AZM, C, CIP, CL, CN, CRO, CXM, FOS, LEV, MEM, NA, OT, S | 15 (8) | 0.78 | 49/70  (70%) |
| 47E | ET, EA, EH, EP, EI | AK, AMC, AML, C, CIP, CL, CN, CRO, DO, FOX, NA, LEV, OT, S | 14 (6) | 0.73 |  |
| 34E | ET, EA, EP | AK, AMC, AML, AZM, NA, DO, FOX, CL, IPM, MEM, FOS, OT, S | 13 (7) | 0.68 |  |
| 51E | ET, EP, EI | AK, AMC, AML, CL, CN, CRO, CXM, DO, FOS, FOX, MEM, OT, S | 13 (6) | 0.68 |  |
| 79E |  | AK, AMC, AML, AZM, CIP, CL, FOS, FOX, LEV, MEM, NA, OT, S | 13 (7) | 0.68 |  |
| 87E | ET, EA, EP | AMC, AML, AZM, CL, CRO, CXM, DO, FOS, FOX, MEM, NA, OT, S | 13 (7) | 0.68 |  |
| 65E | ET, EP | AK, AMC, AML, AZM, CRO, CXM, FOS, FOX, IPM, MEM, OT, S | 12 (6) | 0.63 |  |
| 99E | ET, EP, EI | AK, AMC, AML, CL, CRO, CXM, DO, FOX, MEM, NA, OT, S | 12 (6) | 0.63 |  |
| 56E | ET, EH, EP, EI | AK, AML, CIP, CL, CN, FOX, LEV, MEM, NA, OT, S | 11 (6) | 0.57 |  |
| 82E | ET, EP, EI | AK, AMC, AML, AZM, CL, CRO, CXM, FOX, MEM, OT, S | 11 (5) | 0.57 |  |
| 96E |  | AK, AMC, AML, CIP, CL, DO, FOS, FOX, MEM, S, OT | 11 (7) | 0.57 |  |
| 74E | ET, EA, EH, EP | AK, AML, AZM, CL, CN, FOS, FOX, MEM, NA, OT, S | 11 (7) | 0.57 |  |
| 38E | EP | AK, AML, AZM, CL, CN, DO, FOX, MEM, OT, S | 10 (5) | 0.52 |  |
| 39E | EA, EP, EI | AMC, AML, AZM, CL, CRO, CXM, FOX, MEM, OT, S | 10 (5) | 0.52 |  |
| 48E |  | AK, AMC, AML, CIP, CL, CN, CRO, FOX, MEM, OT, S | 11 (6) | 0.52 |  |
| 59E |  | AMC, AML, AZM, CL, CN, FOS, FOX, MEM, S, OT | 10 (6) | 0.52 |  |
| 61E | ET, EA | AMC, AML, AZM, CL, FOS, FOX, MEM, NA, OT, S | 10 (7) | 0.52 |  |
| 63E | ET, EP | AK, AMC, AML, CL, CXM, FOS, FOX, MEM, S, OT | 10 (6) | 0.52 |  |
| 68E | ET, EA, EP | AK, AML, CIP, CL, CN, CRO, LEV, MEM, NA, S | 10 (5) | 0.52 |  |
| 88E |  | AMC, AML, AZM, CL, CXM, FOS, FOX, MEM, NA, S, OT | 11 (7) | 0.57 |  |
| 40E | ET, EA, EP, EI | AK, AMC, AML, AZM, DO, MEM, NA, OT, S | 09 (5) | 0.47 |  |
| 49E |  | AMC, AML, AZM, CL, CXM, FOS, FOX, MEM, S | 09 (6) | 0.47 |  |
| 83E | ET, EP, EI | AMC, AML, FOX, CXM, FOS, MEM, OT, S | 09 (6) | 0.47 |  |
| 84E |  | AMC, AML, CL, CXM, FOS, FOX, MEM, OT, S | 09 (6) | 0.47 |  |
| 85E | EH, EP, EI | AMC, AML, CL, CRO, CXM, FOS, FOX, MEM, S | 09 (5) | 0.47 |  |
| 97E | ET, EP, EI | AMC, AML, CRO, CXM, FOS, FOX, IPM, MEM, S | 09 (5) | 0.47 |  |
| 13E | ET, EP, EI | AMC, AML, CFM, CL, FOX, IPM, MEM, S | 8 (4) | 0.42 |  |
| 26E |  | AK, AMC, AML, CFM, CL, S, FOX, MEM | 8 (4) | 0.42 |  |
| 66E | EP | AK, AML, CL, CN, MEM, NA, OT, S | 8 (6) | 0.42 |  |
| 67E |  | AK, AMC, AML, CIP, CL, FOS, FOX, MEM | 8 (6) | 0.42 |  |
| 78E |  | AMC, AML, AZM, CL, FOS, FOX, MEM, OT | 8 (5) | 0.42 |  |
| 81E |  | AK, AMC, AML, AZM, CL, FOX, MEM, OT | 08 (5) | 0.42 |  |
| 93E | ET, EI | AK, AML, CL, CN, FOX, IPM, MEM, S | 8 (4) | 0.42 |  |
| 94E | ET, EP, EI | AK, AMC, AML, CL, FOX, IPM, MEM, S | 8 (4) | 0.42 |  |
| 29E | ET, EI | AK, AMC, AML, CIP, FOX, MEM, OT, S | 8 (6) | 0.42 |  |
| 37E | ET, EP, EI | AK, AML, CN, IPM, MEM, OT, S | 7 (4) | 0.36 |  |
| 46E | ET, EA, EH, EP, EI | AML, AZM, CL, FOX, MEM, OT, S | 7 (5) | 0.36 |  |
| 50E |  | AML, CIP, FOS, LEV, MEM, S | 6 (5) | 0.31 |  |
| 71E |  | AMC, AML, CL, FOS, FOX, MEM, OT | 7 (5) | 0.36 |  |
| 89E | ET, EI | AK, AMC, AML, CL, FOX, MEM, S | 7 (4) | 0.36 |  |
| 100E |  | AK, AML, CIP, CL, CN, FOX, S | 7 (4) | 0.36 |  |
| 9E | EA, EH, EP, EI | AK, AMC, AML, MEM, S | 5 (3) | 0.26 |  |
| 33E | ET, EH, EP, EI | AK, AML, AZM, CN, MEM, S | 6 (3) | 0.31 |  |
| 41E |  | AML, AZM, DO, OT, MEM, S | 6 (4) | 0.31 |  |
| 44E |  | AML, CL, FOX, MEM, OT, S | 6 (5) | 0.31 |  |
| 45E | ET, EP, EI | AMC, AML, CL, FOX, MEM, OT | 6 (4) | 0.31 |  |
| 60E | ET, EA, EP, EI | AMC, AML, CL, FOS, FOX, MEM | 6 (4) | 0.31 |  |
| 64E |  | AML, AZM, CIP, CL, OT, MEM | 6 (5) | 0.31 |  |
| 75E | ET, EA, EP, EI | AML, AZM, CL, MEM, OT, S | 6 (5) | 0.31 |  |
| 92E | ET, EP, EI | AMC, AML, CL, FOS, FOX, MEM | 6 (5) | 0.31 |  |
| 17E |  | AML, CRO, CXM, MEM | 4 (3) | 0.21 |  |
| 90E |  | AK, AMC, AML, MEM, S | 5 (3) | 0.26 |  |
| 98E | ET, EP, EI | AK, AMC, AML, CL, MEM | 5 (4) | 0.26 |  |
| 28E |  | AML, CL, CRO, MEM | 4 (3) | 0.21 |  |
| 52E | ET, EP | AML, AZM, MEM, S | 4 (3) | 0.21 |  |
| 73E |  | AML, OT, MEM, S | 4 (4) | 0.21 |  |
| 91E | EP, EI | AMC, AML, MEM, S | 4 (3) | 0.21 |  |
| 77E |  | AML, AZM, FOX, MEM, OT | 5 (4) | 0.26 |  |
| 15E |  | AML, MEM, S | 3 (3) | 0.15 |  |
| 24E |  | AML, CL, MEM | 3 (3) | 0.15 |  |
| 86E | ET, EI | AML, IPM, MEM | 3 (2) | 0.15 |  |
| 2E | ET, EP, EI | AML, S | 2 (2) | 0.105 |  |
| 3E |  | AML, AZM, S | 3 (3) | 0.157 |  |
| 7E | ET, EA, EP, EI | AML, CL | 2 (2) | 0.105 |  |
| 23E | EA, EP, EI | AML, MEM | 2 (2) | 0.105 |  |
| 42E |  | AK, AML, S | 3 (2) | 0.157 |  |
| 70E | EH, EP | AK, AML | 2 (2) | 0.105 |  |
| 8E | ET, EA, EH, EP, EI | AML | 1 (1) | 0.052 |  |
| 16E |  | AML | 1 (1) | 0.152 |  |
| 30E | ET, EP, EI | AML, S | 2 (2) | 0.105 |  |

Enteropathogenic *E. coli,* EP*;* Enteroinvasive *E. coli,* EI*;* Enterotoxigenic *E. coli,* ET*;* Enteroaggregative *E. coli,* EA*;* Enterohemorrhagic *E. coli,* EH
