# Supplementary material for: Migratory Birds Facilitate the Spread of Multidrug‐Resistant Pathogenic Escherichia coli in Tanguar Haor of Bangladesh
Source: Environ Microbiol Rep. 2026 Apr 12;18(2):e70344. doi: 10.1111/1758-2229.70344 (PMC13070580; doi:10.1111/1758-2229.70344)
Supplement: Supplementary file 2 — Table S2: Multidrug‐resistant patterns of the E. coli isolated from the Tanguar Haor in 2024. [file EMI4-18-e70344-s004.docx]

**Supplementary table 2:** Multidrug-resistant patterns of the *E. coli* isolated from the Tanguar haor in 2024

| **Sample ID** | **Presence of Pathotypes** | **Multidrug-Resistance Patterns** | **No. of Resistant Antibiotics (Classes)** | **MAR (a/b)** | **Overall**  **No. of**  **MDR**  **isolates (%)** |
| --- | --- | --- | --- | --- | --- |
| 22B-E |  | AK, AMC, AML, AZM, CIP, CN, CL, CRO, CXM, DO, FOS, FOX, IPM, MEM, NA, OT, S | 17 (7) | 0.89 | 30/30  (100%) |
| 55B-E | EP | AK, AMC, AML, AZM, C, CIP, CL, CN, CRO, CXM, DO, FOX, IPM, MEM, OT, S | 16 (7) | 0.84 |  |
| 17B-E |  | AK, AMC, AML, AZM, CIP, CL, CN, CXM, DO, FOS, FOX, IPM, MEM, NA, OT, S | 16 (7) | 0.84 |  |
| 59B-E |  | AK, AMC, AML, AZM, C, CIP, CL, CN, CXM, FOX, IPM, LEV, MEM, NA, OT, S | 16 (7) | 0.84 |  |
| 21B-E |  | AK, AMC, AML, AZM, CL, CN, CXM, DO, FOS, FOX, IPM, MEM, NA, OT, S | 15 (7) | 0.78 |  |
| 24B-E |  | AK, AMC, AML, AZM, CL, CN, CXM, DO, FOS, FOX, IPM, MEM, NA, OT, S | 15 (7) | 0.78 |  |
| 27B-E |  | AK, AMC, AML, AZM, CL, CIP, CN, CXM, FOS, FOX, IPM, MEM, NA, OT, S | 15 (7) | 0.78 |  |
| 28B-E | EH, EP | AK, AMC, AML, AZM, CIP, CL, CN, CRO, DO, FOS, FOX, IPM, LEV, OT, S | 15 (7) | 0.78 |  |
| 38B-E |  | AK, AMC, AML, AZM, CIP, CL, CN, CRO, CXM, DO, FOS, FOX, NA, OT, S | 15 (7) | 0.78 |  |
| 6B-E |  | AML, AZM, C, CIP, CL, CN, CRO, CXM, FOX, LEV, MEM, NA, OT, S | 14 (7) | 0.73 |  |
| 39B-E |  | AK, AMC, AML, AZM, CL, CN, CRO, CXM, DO, FOX, MEM, NA, OT, S | 14 (6) | 0.73 |  |
| 48B-E |  | AK, AMC, AML, AZM, CIP, CL, CN, CRO, CXM, FOS, FOX, IPM, OT, S | 14 (7) | 0.73 |  |
| SB-1E |  | AMC, AML, AZM, CL, CN, CXM, DO, LEV, FOS, FOX, MEM, OT, S | 13 (7) | 0.68 |  |
| SB-8E | ET, EP | AMC, AML, AZM, CN, CL, CXM, DO, FOS, FOX, IPM, MEM, OT, S | 13 (6) | 0.68 |  |
| 12B-E | ET, EP | AK, AMC, AML, AZM, CIP, CL, DO, FOX, IPM, MEM, NA, OT, S | 13 (6) | 0.68 |  |
| 16B-E |  | AK, AMC, AML, AZM, CIP, CL, CN, CRO, DO, FOX, MEM, OT, S | 13 (6) | 0.68 |  |
| 19B-E |  | AK, AMC, AML, AZM, CL, CN, FOS, FOX, IPM, MEM, NA, OT, S | 13 (7) | 0.68 |  |
| 50B-E |  | AK, AMC, AML, AZM, CIP, CL, CN, CXM, FOX, IPM, NA, OT, S | 13 (7) | 0.68 |  |
| SB-9E |  | AK, AMC, AML, CL, CN, CXM, FOS, FOX, IPM, MEM, OT, S | 12 (6) | 0.63 |  |
| 20B-E |  | AK, AMC, AML, AZM, CL, CN, CRO, CXM, DO, FOS, FOX, NA | 12 (7) | 0.63 |  |
| 30B-E |  | AK, AMC, AML, AZM, CL, CN, CXM, FOS, MEM, NA, OT, S | 12 (7) | 0.63 |  |
| 32B-E |  | AK, AMC, AML, AZM, CL, CN, CXM, DO, FOX, MEM, OT, S | 12 (5) | 0.63 |  |
| 113B-E | ET | AK, AML, AZM, CIP, CL, CN, DO, FOX, MEM, NA, OT, S | 12 (6) | 0.63 |  |
| SB-2E | ET, EP | AK, AMC, AML, AZM, CL, CN, DO, FOX, MEM, OT, S | 11 (6) | 0.57 |  |
| SB-5E |  | AK, AMC, AML, CIP, CL, CN, CXM, FOX, NA, OT, S | 11 (5) | 0.57 |  |
| 35B-E | ET | AK, AMC, AML, CL, CN, CXM, FOS, FOX, MEM, OT, S | 11 (6) | 0.57 |  |
| 98B-E |  | AK, AML, AZM, CL, CN, CRO, FOX, MEM, NA, OT, S | 11 (6) | 0.57 |  |
| SB-11E |  | AK, AMC, AML, CIP, CN, FOX, MEM, NA, OT, S | 10 (6) | 0.52 |  |
| 58B-E |  | AK, AML, C, CN, CRO, DO, FOX, NA, OT, S | 10 (6) | 0.52 |  |
| 13B-E |  | AK, AML, AMC, CL, CN, MEM, NA, OT, S | 09 (6) | 0.47 |  |

Enteropathogenic *E. coli,* EPEC*;* Enterotoxigenic *E. coli,* ETEC*;* Enterohemorrhagic *E. coli,* EHEC
