# Supplementary material for: Migratory Birds Facilitate the Spread of Multidrug‐Resistant Pathogenic Escherichia coli in Tanguar Haor of Bangladesh
Source: Environ Microbiol Rep. 2026 Apr 12;18(2):e70344. doi: 10.1111/1758-2229.70344 (PMC13070580; doi:10.1111/1758-2229.70344)
Supplement: Supplementary file 3 — Table S3: Pearson correlation coefficient of antibiotics and antibiotics correlation with 95% significance in E. coli isolates from Tanguar Haor‐2023. [file EMI4-18-e70344-s002.docx]

**Supplementary table 3:** Pearson correlation coefficient of antibiotics and antibiotics correlation with 95% significance in E. coli isolates from Tanguar haor-2023

|  | | **AML** | **AMC** | **NA** | **CIP** | **LEV** | **C** | **AK** | **CN** | **S** | **OT** | **DO** | **CRO** | **FOX** | **CL** | **CXM** | **AZM** | **IPM** | **MEM** | **FOS** |
| --- | --- | --- | --- | --- | --- | --- | --- | --- | --- | --- | --- | --- | --- | --- | --- | --- | --- | --- | --- | --- |
| **AML** | Pearson Correlation | 1 |  |  |  |  |  |  |  |  |  |  |  |  |  |  |  |  |  |  |
|  | Sig. (2-tailed) |  |  |  |  |  |  |  |  |  |  |  |  |  |  |  |  |  |  |  |
| **AMC** | Pearson Correlation | .584^**^ | 1 |  |  |  |  |  |  |  |  |  |  |  |  |  |  |  |  |  |
|  | Sig. (2-tailed) | .000 |  |  |  |  |  |  |  |  |  |  |  |  |  |  |  |  |  |  |
| **NA** | Pearson Correlation | .062 | .118 | 1 |  |  |  |  |  |  |  |  |  |  |  |  |  |  |  |  |
|  | Sig. (2-tailed) | .613 | .330 |  |  |  |  |  |  |  |  |  |  |  |  |  |  |  |  |  |
| **CIP** | Pearson Correlation | .119 | .017 | .644^**^ | 1 |  |  |  |  |  |  |  |  |  |  |  |  |  |  |  |
|  | Sig. (2-tailed) | .327 | .886 | .000 |  |  |  |  |  |  |  |  |  |  |  |  |  |  |  |  |
| **LEV** | Pearson Correlation | .086 | .092 | .667^**^ | .819^**^ | 1 |  |  |  |  |  |  |  |  |  |  |  |  |  |  |
|  | Sig. (2-tailed) | .480 | .451 | .000 | .000 |  |  |  |  |  |  |  |  |  |  |  |  |  |  |  |
| **C** | Pearson Correlation | -.001 | -.043 | .430^**^ | .663^**^ | .486^**^ | 1 |  |  |  |  |  |  |  |  |  |  |  |  |  |
|  | Sig. (2-tailed) | .996 | .724 | .000 | .000 | .000 |  |  |  |  |  |  |  |  |  |  |  |  |  |  |
| **AK** | Pearson Correlation | .097 | -.095 | .346^**^ | .388^**^ | .359^**^ | .357^**^ | 1 |  |  |  |  |  |  |  |  |  |  |  |  |
|  | Sig. (2-tailed) | .422 | .436 | .003 | .001 | .002 | .002 |  |  |  |  |  |  |  |  |  |  |  |  |  |
| **CN** | Pearson Correlation | .154 | -.038 | .470^**^ | .695^**^ | .547^**^ | .685^**^ | .531^**^ | 1 |  |  |  |  |  |  |  |  |  |  |  |
|  | Sig. (2-tailed) | .203 | .756 | .000 | .000 | .000 | .000 | .000 |  |  |  |  |  |  |  |  |  |  |  |  |
| **S** | Pearson Correlation | .422^**^ | .183 | .182 | .270^*^ | .298^*^ | .298^*^ | .137 | .353^**^ | 1 |  |  |  |  |  |  |  |  |  |  |
|  | Sig. (2-tailed) | .000 | .129 | .131 | .024 | .012 | .012 | .258 | .003 |  |  |  |  |  |  |  |  |  |  |  |
| **OT** | Pearson Correlation | .568^**^ | .164 | .454^**^ | .336^**^ | .299^*^ | .118 | .238^*^ | .292^*^ | .268^*^ | 1 |  |  |  |  |  |  |  |  |  |
|  | Sig. (2-tailed) | .000 | .176 | .000 | .004 | .012 | .332 | .047 | .014 | .025 |  |  |  |  |  |  |  |  |  |  |
| **DO** | Pearson Correlation | .434^**^ | .253^*^ | .428^**^ | .224 | .273^*^ | .161 | .175 | .238^*^ | .311^**^ | .676^**^ | 1 |  |  |  |  |  |  |  |  |
|  | Sig. (2-tailed) | .000 | .034 | .000 | .062 | .022 | .183 | .148 | .047 | .009 | .000 |  |  |  |  |  |  |  |  |  |
| **CRO** | Pearson Correlation | .146 | .174 | .315^**^ | .465^**^ | .394^**^ | .338^**^ | .194 | .460^**^ | .335^**^ | .157 | .212 | 1 |  |  |  |  |  |  |  |
|  | Sig. (2-tailed) | .229 | .150 | .008 | .000 | .001 | .004 | .108 | .000 | .005 | .196 | .078 |  |  |  |  |  |  |  |  |
| **FOX** | Pearson Correlation | .513^**^ | .590^**^ | .223 | .210 | .280^*^ | .057 | .223 | .177 | .240^*^ | .424^**^ | .241^*^ | .260^*^ | 1 |  |  |  |  |  |  |
|  | Sig. (2-tailed) | .000 | .000 | .063 | .081 | .019 | .642 | .063 | .142 | .045 | .000 | .045 | .030 |  |  |  |  |  |  |  |
| **CL** | Pearson Correlation | .545^**^ | .481^**^ | .185 | .241^*^ | .238^*^ | .115 | -.081 | .216 | .225 | .321^**^ | .187 | .287^*^ | .598^**^ | 1 |  |  |  |  |  |
|  | Sig. (2-tailed) | .000 | .000 | .125 | .045 | .048 | .341 | .505 | .073 | .061 | .007 | .122 | .016 | .000 |  |  |  |  |  |  |
| **CXM** | Pearson Correlation | .173 | .336^**^ | .210 | .105 | .144 | .143 | -.102 | -.002 | .224 | .199 | .240^*^ | .621^**^ | .282^*^ | .218 | 1 |  |  |  |  |
|  | Sig. (2-tailed) | .151 | .005 | .082 | .385 | .234 | .239 | .399 | .986 | .063 | .099 | .045 | .000 | .018 | .070 |  |  |  |  |  |
| **AZM** | Pearson Correlation | .178 | .035 | .370^**^ | .174 | .113 | .252^*^ | .062 | .051 | .088 | .315^**^ | .188 | .063 | .207 | .294^*^ | .240^*^ | 1 |  |  |  |
|  | Sig. (2-tailed) | .141 | .772 | .002 | .151 | .352 | .035 | .608 | .674 | .471 | .008 | .119 | .606 | .085 | .014 | .045 |  |  |  |  |
| **IPM** | Pearson Correlation | .146 | .215 | .111 | .095 | .189 | .154 | .039 | .010 | .172 | -.009 | .097 | .185 | .308^**^ | .115 | .201 | .163 | 1 |  |  |
|  | Sig. (2-tailed) | .226 | .074 | .360 | .434 | .117 | .202 | .751 | .936 | .154 | .941 | .427 | .126 | .009 | .343 | .096 | .177 |  |  |  |
| **MEM** | Pearson Correlation | .070 | .339^**^ | .155 | .099 | .169 | .151 | .063 | .087 | .029 | .081 | -.006 | .141 | .269^*^ | .146 | .316^**^ | .232 | .202 | 1 |  |
|  | Sig. (2-tailed) | .565 | .004 | .201 | .413 | .163 | .211 | .603 | .474 | .812 | .506 | .961 | .246 | .024 | .229 | .008 | .053 | .093 |  |  |
| **FOS** | Pearson Correlation | .460^**^ | .571^**^ | .285^*^ | .194 | .300^*^ | .046 | .084 | .099 | .191 | .195 | .138 | .174 | .526^**^ | .492^**^ | .404^**^ | .269^*^ | .148 | .424^**^ | 1 |
|  | Sig. (2-tailed) | .000 | .000 | .017 | .108 | .011 | .704 | .487 | .414 | .113 | .107 | .254 | .149 | .000 | .000 | .001 | .025 | .220 | .000 |  |

AML = Amoxicillin; AMC = Amoxicillin + Clavulanic acid; NA = Nalidixic acid; CIP = Ciprofloxacin; LEV = Levofloxacin; C = Chloramphenicol; AK = Amikacin; CN = Gentamicin; S = Streptomycin; OT = Oxytetracycline; DO = Doxycycline; CRO = Ceftriaxone; FOX = Cefoxitin; CL = Cephalexin; CXM = Cefuroxime; AZM = Azithromycin; IPM = Imipenem; MEM = Meropenem; FOS = Fosfomycin

| **. Correlation is significant at the 0.01 level (2-tailed). |
| --- |
| *. Correlation is significant at the 0.05 level (2-tailed). |
| ***. Correlation at 0.001(2-tailed) |
