# Supplementary material for: Migratory Birds Facilitate the Spread of Multidrug‐Resistant Pathogenic Escherichia coli in Tanguar Haor of Bangladesh
Source: Environ Microbiol Rep. 2026 Apr 12;18(2):e70344. doi: 10.1111/1758-2229.70344 (PMC13070580; doi:10.1111/1758-2229.70344)
Supplement: Supplementary file 4 — Table S4: Pearson correlation coefficient of antibiotics and antibiotics correlation with 95% significance in E. coli isolates from Tanguar Haor‐2024. [file EMI4-18-e70344-s003.docx]

**Supplementary table 4:** Pearson correlation coefficient of antibiotics and antibiotics correlation with 95% significance in E. coli isolates from Tanguar haor-2024

|  | | **AML** | **AMC** | **NA** | **CIP** | **LEV** | **C** | **AK** | **CN** | **S** | **OT** | **DO** | **CRO** | **FOX** | **CL** | **CXM** | **AZM** | **IPM** | **MEM** | **FOS** |
| --- | --- | --- | --- | --- | --- | --- | --- | --- | --- | --- | --- | --- | --- | --- | --- | --- | --- | --- | --- | --- |
| **AML** | Pearson Correlation | 1 |  |  |  |  |  |  |  |  |  |  |  |  |  |  |  |  |  |  |
|  | Sig. (2-tailed) |  |  |  |  |  |  |  |  |  |  |  |  |  |  |  |  |  |  |  |
| **AMC** | Pearson Correlation | .837^**^ | 1 |  |  |  |  |  |  |  |  |  |  |  |  |  |  |  |  |  |
|  | Sig. (2-tailed) | .000 |  |  |  |  |  |  |  |  |  |  |  |  |  |  |  |  |  |  |
| **NA** | Pearson Correlation | .046 | -.141 | 1 |  |  |  |  |  |  |  |  |  |  |  |  |  |  |  |  |
|  | Sig. (2-tailed) | .809 | .456 |  |  |  |  |  |  |  |  |  |  |  |  |  |  |  |  |  |
| **CIP** | Pearson Correlation | .074 | -.072 | .139 | 1 |  |  |  |  |  |  |  |  |  |  |  |  |  |  |  |
|  | Sig. (2-tailed) | .696 | .707 | .462 |  |  |  |  |  |  |  |  |  |  |  |  |  |  |  |  |
| **LEV** | Pearson Correlation | .196 | .038 | -.099 | .660^**^ | 1 |  |  |  |  |  |  |  |  |  |  |  |  |  |  |
|  | Sig. (2-tailed) | .300 | .841 | .602 | .000 |  |  |  |  |  |  |  |  |  |  |  |  |  |  |  |
| **C** | Pearson Correlation | .062 | -.251 | .264 | .296 | .157 | 1 |  |  |  |  |  |  |  |  |  |  |  |  |  |
|  | Sig. (2-tailed) | .746 | .181 | .159 | .112 | .407 |  |  |  |  |  |  |  |  |  |  |  |  |  |  |
| **AK** | Pearson Correlation | -.064 | -.189 | .342 | -.261 | -.377^*^ | .103 | 1 |  |  |  |  |  |  |  |  |  |  |  |  |
|  | Sig. (2-tailed) | .737 | .316 | .064 | .164 | .040 | .590 |  |  |  |  |  |  |  |  |  |  |  |  |  |
| **CN** | Pearson Correlation | .149 | .020 | .093 | -.235 | -.067 | .241 | .230 | 1 |  |  |  |  |  |  |  |  |  |  |  |
|  | Sig. (2-tailed) | .431 | .917 | .626 | .211 | .725 | .199 | .221 |  |  |  |  |  |  |  |  |  |  |  |  |
| **S** | Pearson Correlation | .021 | -.159 | .251 | .349 | -.043 | .303 | .232 | -.150 | 1 |  |  |  |  |  |  |  |  |  |  |
|  | Sig. (2-tailed) | .911 | .403 | .181 | .059 | .820 | .103 | .216 | .429 |  |  |  |  |  |  |  |  |  |  |  |
| **OT** | Pearson Correlation | -.120 | -.160 | -.225 | .183 | .032 | .146 | .059 | -.118 | .212 | 1 |  |  |  |  |  |  |  |  |  |
|  | Sig. (2-tailed) | .528 | .398 | .232 | .334 | .868 | .441 | .755 | .535 | .261 |  |  |  |  |  |  |  |  |  |  |
| **DO** | Pearson Correlation | .237 | .161 | .020 | .096 | .236 | .133 | -.226 | -.192 | .120 | -.042 | 1 |  |  |  |  |  |  |  |  |
|  | Sig. (2-tailed) | .207 | .394 | .917 | .613 | .209 | .483 | .230 | .308 | .527 | .827 |  |  |  |  |  |  |  |  |  |
| **CRO** | Pearson Correlation | -.101 | -.274 | .141 | .437^*^ | .378^*^ | .212 | -.013 | -.045 | .053 | -.379^*^ | .336 | 1 |  |  |  |  |  |  |  |
|  | Sig. (2-tailed) | .595 | .143 | .456 | .016 | .039 | .262 | .946 | .815 | .782 | .039 | .070 |  |  |  |  |  |  |  |  |
| **FOX** | Pearson Correlation | .353 | .212 | -.132 | .385^*^ | .232 | .204 | -.093 | .324 | .059 | .079 | .020 | .232 | 1 |  |  |  |  |  |  |
|  | Sig. (2-tailed) | .056 | .261 | .486 | .035 | .216 | .280 | .627 | .081 | .758 | .679 | .917 | .217 |  |  |  |  |  |  |  |
| **CL** | Pearson Correlation | .275 | .222 | -.162 | -.008 | -.039 | -.153 | .175 | .201 | .151 | -.069 | .040 | .145 | .621^**^ | 1 |  |  |  |  |  |
|  | Sig. (2-tailed) | .141 | .238 | .393 | .966 | .836 | .420 | .354 | .288 | .426 | .715 | .832 | .445 | .000 |  |  |  |  |  |  |
| **CXM** | Pearson Correlation | .100 | .123 | .184 | -.008 | -.006 | -.265 | .069 | .077 | .221 | -.398^*^ | .071 | .165 | .062 | .493^**^ | 1 |  |  |  |  |
|  | Sig. (2-tailed) | .599 | .518 | .330 | .965 | .977 | .157 | .719 | .685 | .240 | .029 | .709 | .384 | .745 | .006 |  |  |  |  |  |
| **AZM** | Pearson Correlation | .283 | .214 | -.040 | .232 | .357 | -.189 | .155 | -.035 | .229 | -.062 | .177 | .232 | .257 | .435^*^ | .417^*^ | 1 |  |  |  |
|  | Sig. (2-tailed) | .130 | .255 | .834 | .217 | .053 | .318 | .414 | .853 | .223 | .743 | .350 | .217 | .171 | .016 | .022 |  |  |  |  |
| **IPM** | Pearson Correlation | .282 | .092 | -.073 | .132 | .126 | -.008 | .115 | .259 | .105 | .095 | -.076 | .031 | .625^**^ | .562^**^ | .222 | .318 | 1 |  |  |
|  | Sig. (2-tailed) | .132 | .630 | .703 | .485 | .506 | .966 | .546 | .167 | .582 | .619 | .691 | .872 | .000 | .001 | .239 | .087 |  |  |  |
| **MEM** | Pearson Correlation | .194 | .106 | -.080 | .163 | .089 | -.111 | -.035 | -.118 | .202 | .265 | .111 | -.157 | .181 | .302 | .229 | .410^*^ | .143 | 1 |  |
|  | Sig. (2-tailed) | .304 | .576 | .673 | .390 | .640 | .560 | .854 | .535 | .285 | .157 | .560 | .407 | .339 | .105 | .224 | .024 | .451 |  |  |
| **FOS** | Pearson Correlation | .036 | .099 | .006 | -.272 | -.120 | -.247 | .078 | .303 | -.223 | -.393^*^ | -.297 | .070 | .149 | .161 | .267 | .178 | .345 | -.151 | 1 |
|  | Sig. (2-tailed) | .851 | .602 | .975 | .145 | .529 | .188 | .683 | .104 | .236 | .032 | .110 | .712 | .431 | .395 | .153 | .345 | .062 | .425 |  |

AML = Amoxicillin; AMC = Amoxicillin + Clavulanic acid; NA = Nalidixic acid; CIP = Ciprofloxacin; LEV = Levofloxacin; C = Chloramphenicol; AK = Amikacin; CN = Gentamicin; S = Streptomycin; OT = Oxytetracycline; DO = Doxycycline; CRO = Ceftriaxone; FOX = Cefoxitin; CL = Cephalexin; CXM = Cefuroxime; AZM = Azithromycin; IPM = Imipenem; MEM = Meropenem; FOS = Fosfomycin

| **. Correlation is significant at the 0.01 level (2-tailed). |
| --- |
| *. Correlation is significant at the 0.05 level (2-tailed). |
| ***. Correlation at 0.001(2-tailed) |
